# Genetic variability of human papillomavirus type 39 based on E6, E7 and L1 genes in Southwest China

**DOI:** 10.1186/s12985-021-01528-w

**Published:** 2021-04-08

**Authors:** Jiaoyu He, Tianjun Li, Youliang Wang, Zhilin Song, Qiufu Li, Yiran Liu, Yanru Cui, Siyu Ma, Junhang Deng, Xia Wei, Xianping Ding

**Affiliations:** 1 Key Laboratory of Bio-Resources and Eco-Environment of Ministry of Education, College of Life Sciences, Sichuan University, Chengdu, 610065 Sichuan People’s Republic of China; 2 Bio-Resource Research and Utilization Joint Key Laboratory of Sichuan and Chongqing, Chongqing Nanchuan Biotechnology Research Institute, Chongqing, Sichuan People’s Republic of China; 3 The People’s Hospital of Pengzhou, Pengzhou, Sichuan People’s Republic of China; 4 Institute of Medical Genetics, College of Life Sciences, Sichuan University, Chengdu, 610064 China

**Keywords:** HPV39, Genetic variability, E6, E7 and L1 genes, Cervical cancer, Southwest china, Lineage phylogeny, protein structure

## Abstract

**Background:**

Human papillomavirus type 39 associated with genital intraepithelial neoplasia and invasive cancers, has a high prevalence in Southwest China. HPV E6, E7 are two main papillomavirus oncoproteins, closely relate to the function of HPV immortalization, cell transformation, and carcinogenesis. L1 is the major capsid protein, can reflect the replication status of the virus in cells and the progression of cervical lesions. The purpose of this study is to reveal the prevalence of HPV 39 and the genetic polymorphisms of HPV39 based on *E6, E7* and *L1* gene in southwest China.

**Methods:**

Cell samples were collected by cervical scraped for HPV detecting and typing, and HPV39 positive samples were selected out. Important *E6, E7* and *L1* genes of HPV39 were sequenced and analyzed for the study of HPV39 genetic polymorphisms. Phylogenetic trees were constructed by Maximum-likelihood and Kimura 2-parameters methods in Molecular Evolutionary Genetics Analysis version 6.0. The selection pressures of *E6, E7* and *L1* genes were estimated by Datamonkey web server. The secondary and three-dimensional structure of HPV39 E6, E7 proteins were created by sopma server and SWISS-MODEL software.

**Results:**

344 HPV39 positive samples were selected from 5718 HPV positive cell samples. Among HPV39 *E6-E7* sequences, 20 single nucleotide mutations were detected, including 10 non-synonymous and 10 synonymous mutations; 26 single nucleotide mutations were detected in HPV39 *L1* sequences, including 7 non-synonymous and 19 synonymous mutations respectively. 11 novel variants of HPV39 *E6-E7* (5 in *E6* and 6 in *E7*) and 14 novel variants of HPV39 *L1* were identified in this study. A-branch was the most frequent HPV39 lineage in southwest China during our investigation. Selective pressure analysis showed that codon sites 26, 87, 151 in *E6* and 75, 180, 222, 272, 284, 346, 356 in *L1* were positively selected sites, as well as codon sites 45, 138, 309, 381 were negative selection sites in *L1* gene, *E7* has neither positive selection sites nor negative selection sites. A certain degree of secondary and three-dimensional structure dislocation was existed due to the non-synonymous mutations.

**Conclusions:**

Amino acid substitution affected the secondary and three-dimensional structure of HPV39, and resulting in the differences of carcinogenic potential and biological functions as well as the immune response due to the antigen epitopes difference, the antigen epitopes with stronger adaptability in Southwest will be screened out based on the above research results for the later vaccine development. And gene polymorphism of HPV39 in Southwest China may improve the effectiveness of clinical test and vaccine design, specifically for women in Southwest China.

## Introduction

Cervical cancer (CC) is the fourth leading cancer in women worldwide. The incidence of CC in developing countries is almost as twice as in developed countries, 17.8% and 9.0% respectively [1]. According to the report by the International Agency for Research on Cancer in 2018, the CC infection rate among Chinese women is 10.7 per 100,000, just below the global rate of 13.1 per 100,000, and increasing, that due to the neglect of screening [2]. Obviously, this cancer has seriously threatened the life and health of Chinese women.

HPV is a small, non-enveloped and double stranded DNA (dsDNA) virus, approximately 8,000 base pairs (bp) which contains early region (E6, E7, E1, E2, E4, and E5) and late region (L1, L2) [3]. High-risk human papilloma virus (HR-HPV) persistent infection is the leading cause of CC. E6 and E7 can inhibit the tumor suppressor pathways of p53 and pRb (retinoblastoma) respectively, that resulting in immortalization, cell transformation, and carcinogenesis, are two main papillomavirus oncoproteins [4–7]. L1 is the major capsid protein, which can reflect the replication status of virus in cells, the detection of L1 capsid protein expression can predict the progression and degeneration of cervical lesions [8].

More than 200 HPV types had been recognized by the International HPV Reference Center [9]. A large number of epidemiological and laboratory research data show that 12 h-HPV are closely related to the occurrence of malignant lesions, such as cervical cancer, including HPV16, 18, 31, 33, 35, 39, 45, 51, 52, 56, 58, and 59, and they mainly belong to the α-9 (HPV16-related) and α-7 (HPV18-related) genus based on the principle of evolutionary classification [10, 11].

**Fig. 1:**
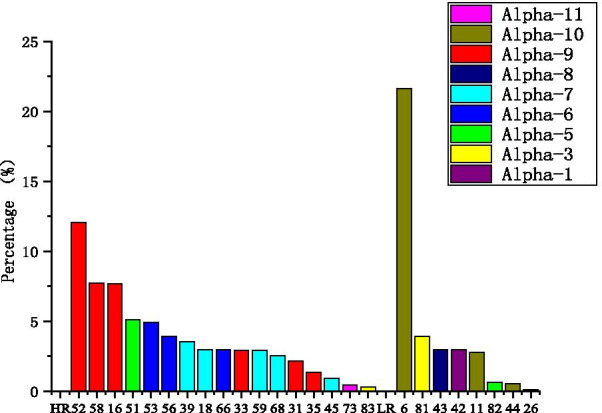
The detection rate of different genus and different subtype HPV in all positive samples

Globally, HPV16 and HPV18 are the most common HR-HPV, persistence infection associated with those causing 70% of CC and pre-cancerous cervical lesions [12]. HPV39 belongs to α-7, has no available commercial vaccines, due to its low infection rate [13]. Nevertheless, the infection rate of different HPV genotypes varies in different geographical regions and different ages [1]. For example, in southern China, HPV infections occurs mainly in women under 30 years old, and HPV52, HPV16, HPV58, HPV18 and HPV39 are the most common HR-HPV subtypes [14]. In western China, HPV16, HPV52, HPV58, HPV33 and HPV18 are the most prevalence HR-HPV subtypes among patients with cervical intraepithelial neoplasia (CIN), HPV39 ranking eighth [15]. In our research, the detection rate of HPV39 is higher than that of HPV18 (Fig. 1). Nowadays, HPV18 has been extensively studied. While, the data available on HPV39 is still limited, especially in China. Therefore, the genetic polymorphisms, lineage phylogeny and positive selections of HPV39 based on *E6, E7* and *L1* genes in southwest China from a large amount of samples are sorely needed for detection of HPV-related diseases and development of HPV vaccine, as well as increased the attention to the China CC related α-7 HPV (HPV18, 39, 45, 59, 68, 70, 85 and 97).

## Materials and methods

### Samples resource

23,054 specimens were randomly collected from January 2015 to August 2019 in the Sichuan Reproductive Health Research Center Affiliated Hospital, Chengdu Song zi niao Sterility Hospital, Infertility Hospital Affiliated to Chengdu Medical College, Chengdu Jinsha hospital, and Angel Women's and Children's Hospital. The cervical swabs were placed in − 20℃ antiseptic buffer for HPV detection and typing. The study was ethically approved by the Education and Research Committee and Ethics Committee of Sichuan University, Sichuan, China. All data was used with the consent of HPV-tested patients.

### Genomic DNA extraction and HPV typing

The viral genomic DNA was extracted by the HEALTH Nucleic Acid Extraction Kit (Ningbo, Zhejiang) and genotyped by 3500DX Genetic Analyzer (Ningbo, Zhejiang), according to the manufacturer's instructions. The gene analyzer can classify 17 h/probable HR-HPV types (16, 18, 31, 33,35, 39, 45, 51, 52, 53, 56, 58, 59, 66, 68, 73, 83) and 8 LR-HPV types (6, 11, 26, 42, 43, 44, 81and 82) based on capillary electrophoresis. 3,087 specimens belonging to α-7 (18, 39, 45, 59, 68) and α-9 (16, 31, 33, 35, 52, 58) were detected, of which 344 were HPV39. The extracted DNA was saved at -20℃ for follow-up experiments.

### PCR amplification and Variant identification

The primers of HPV39 *E6, E7* and *L1* were designed by PRIMER 5.0 based on the HPV39 reference sequences (GeneBank number: M62849) (Table 1) and synthesized by TSINGKE (Chengdu, China). 5 µl HPV39 DNA, 13.1 µl ddH_2_O, 1 µl primers, 0.4 µl transtaq DNA polymerase, 2.5 µl dNTPS, and 3 µl buffer form the PCR reaction system. PCR conditions were: 95 °C for 6 min, 34 cycles according to the following protocol: denaturation at 94 °C for 45 s, annealing at 57 °C for *E6, E7* and *L1* for 50 s and elongation at 72 °C for 60 s. The PCR products were visualized by gel electrophoresis in 2% agarose gel (Sangon Biotech Co., Ltd.). The target products of *E6, E7* and *L1* were purified and sequenced by TSINGKE (Chengdu, China). The complete alignment results of the successfully amplified 306 *E6-E7* and 298 *L1* sequences were all arranged in Tables 2 and 3 respectively.

**Table 1:** The primers of HPV39 *E6/E7/L1*

Gene	Primer sequences	Primer location (nt)	Amplification length (bp)	Annealing temperature (°C)
*E6*	F: 5′-GGGAGTAACCGAAAACGGTC-3’	36–55	821	57
	R: 5′-GTTGTCGCAGAGTATCCCGT-3’	856–837		
*E7*	F: 5′-GGACCGCAGACTAACACGAA-3’	187–206	613	57
	R: 5′-CCCTTTGGGCCTCTTGCATA-3’	799–780		
*L1-1*	F: 5′-GCTCACTACCTTCTGTGGCTT-3’	105–125	1059	57
	R: 5′-ACCCACCATACCACCACGAT-3’	1163–1144		
*L1-2*	F: 5′-ATTGGGGAGCACTGGGGTAA-3’	72–91	1332	56
	R: 5′-ACTTCGGTCGCCACAAAATG-3’	1403–1384

**Table 2:** Nucleotide mutations and amino acid substitution in HPV39 *E6/E7*

Sequence	*E6* base mutation site								*E7* base mutation site											n = 306
	184	205	211	289	366	487	558	559	596	633	634	762	792	819	820	851	853	858	862	
M62849	G	C	C	A	C	T	T	A	G	T	T	T	G	G	C	G	C	A	C	
39PE01	–	–	–	–	–	–	–	–	–	–	–	–	C	–	–	–	–	–	–	24
39PE02	–	–	–	–	–	–	C	–	–	–	–	–	–	–	–	–	G	–	–	35
39PE03	–	T	–	–	–	–	C	–	–	–	–	–	–	–	–	–	G	–	–	18
39PE04	T	–	–	T	G	C	–	G	–	–	C	A	–	–	T	A	–	–	A	17
39PE05	–	–	–	–	–	–	–	–	A	–	–	–	–	–	–	–	–	–	–	23
39PE06	–	–	T	–	–	–	–	–	–	–	–	–	–	–	–	–	–	–	–	46
39PE07	–	–	–	–	–	–	–	–	–	–	–		C	A	–	–	–	–		25
39PE08	–	–	–	–	–	–	–	–	–	–	–	–	–	–	–	–	–	–	–	12
39PE09	–	–	–	–	–	–	–	–	–	–	–	–	–	–	–	–	–	–	A	14
39PE10	–	–	–	–	–	–	–	–	–	G	–	–	–	–	–	–	G	–	–	28
39PE11	–	–	–	–	–	–	–	–	–	–	–	–	–	–	–	–	–	T	–	29
39PE12	–	–	–	–	–	–	G	–	–	–	–	–	C	–	–	–	–	–	–	14
39PE13	–	–	–	–	–	–	G	–	A	–	–	–	–	–	–	–	–	–	–	21
Amino acid mutation	Q26H	V	C	P	A87G	F	L151P/R	L	R2H	D14E	L	D57E	S	Q	L	R87Q	Q88E	L	Q91K

**Table 3:** Nucleotide mutations and amino acid substitution in HPV 39 *L1*

Sequence	*L1* base mutation site																										n = 298
	5777	5865	6056	6134	6180	6257	6306	6380	6428	6456	6479	6493	6500	6506	6569	6638	6668	6679	6710	6785	6854	6869	6903	6956	6974	7064	
M62849	A	G	C	T	T	A	G	C	T	G	A	A	T	T	T	T	A	C	T	C	T	A	C	A	A	C	
39PL01	–	–	T	–	–	G	–	–	–	–	–	–	–	–	–	A	–	–	–	–	–	–	T	–	–	–	25
39PL02	G	–	–	–	–	–	–	–	–	–	–	–	–	–	–	A	–	–	–	–	–	–	T	–	–	–	36
39PL03	G	–	–	–	–	–	–	–	–	–	–	–	–	–	G	A	–	–	–	A	C	–	T	–	–	–	20
39PL04	–	–	–	C	C	–	–	T	–	C	–	C	–	G	–	–	C	G	G	A	–	G	T	–	G	A	16
39PL05	–	–	T	–	–	–	–	–	–	–	–	–	–	–	G	A	–	–	–	A	C	–	T	–	–	–	23
39PL06	G	A	–	–	–	–	–	–	–	–	–	–	–	–	–	A	–	–	–	–	–	–	T	–	–	–	29
39PL07	–	–	–	–	–	–	–	–	–	–	–	–	–	–	–	A	–	–	–	A	C	–	T	–	–	–	27
39PL08	G	A	–	–	–	–	–	–	–	–	–	–	–	–	–	A	–	–	–	A	C	–	T	–	–	–	14
39PL09	G	–	–	–	–	–	–	–	–	–	–	–	–	–	–	A	–	–	–	A	C	–	T	–	–	–	17
39PL10	–	–	T	–	–	–	–	–	–	–	–	–	–	–	G	A	–	–	–	–	–	–	T	–	–	–	8
39PL11	G	A	–	–	–	–	–	–	–	–	G	–	–	–	–	A	–	–	–	A	C	–	T	G	–	–	9
39PL12	–	–	–	–	–	–	–	–	C	–	–	–	–	–	–	A	–	–	–	–	–	–	T	–	–	–	18
39PL13	–	–	–	–	–	–	–	–	–	–	–	–	–	–	–	A	–	–	–	–	–	–	T	–	–	–	12
39PL14	–	–	–	–	–	–	–	–	–	–	–	–	C	–	–	–	–	–	–	–	–	–	T	–	–	–	27
39PL15	–	–	T	–	–	–	–	–	–	–	–	–	–	–	–	A	–	–	–	–	–	–	T	–	–	–	11
39PL16	–	–	T	–	–	–	A	–	–	–	–	–	–	–	G	A	–	–	–	–	–	–	T	–	–	–	6
Amino acid mutation	V	V75M	T	I	S180P	G	D222N	F	R	A272P	T	N284T	G	S	P	V	T	S346C	D356E	V	F	P	L	K	L	V	

### Sequence analysis

#### Genetic polymorphisms and phylogenetic analysis of HPV39 *E6/E7/L1* genes

The *E6, E7* and *L1* sequences obtained in this study were aligned and compared to M62849 by MEGA 6 software. Nucleotide sequences were converted to amino acid sequence by MEGA 6, in order to determine the amino acid changes caused by nucleotide changes. Maximum-likelihood trees for HPV39 isolates were constructed by 807 bp *E6-E7* and 1518 bp *L1* nucleotide sequences respectively with Kimura’s two-parameter model in MEGA 6. The data was bootstrap resampled 500 times for tree topology evaluation.

#### Selective pressure analysis of HPV39 *E6/E7/L1* genes

The selection pressure of HPV39 *E6, E7* and *L1* were predicted by Datamokey online analysis software [16, 17]. The normalized posterior mean of the dN-dS difference and the Bayesian posterior probability (dN > dS) of the positive selection for each codon position were obtained. A Bayes factor greater than 50 indicates that a site is positively selected.

#### The secondary and three-dimensional structure of HPV39 E6/E7 protein

The secondary structure of main carcinogenic protein HPV E6/E7 reference sequence and the amino acid sequence with mutation were predicted by sopma server (self-optimized prediction method) [18], to analyze whether the non-synonymous mutation cause changes in the protein secondary structure or not. Sopma is a server that uses five independent methods (Levin homologous prediction method, gor method, dual prediction method, PhD method and sopma method) to predict and synthesize a "consistent prediction result".

The three-dimensional spatial structure formed by the interaction between secondary structure. The three-dimensional structure of HPV39 E6/E7 reference and mutation sequence were constructed from scratch to understand the effect of non-synonymous mutation. I-tasser (iterative thread optimization) [19] is a hierarchical method for protein structure and function prediction, it identifies the structure template from PDB by means of lomets, and constructs the full-length atom model by segment assembly simulation based on iterative template, then, the three-dimensional model is reprogrammed into thread through protein functional database biolip to get the target protein sequence, thirdly optimized prediction model and minimized energy by Swiss-Pdb Viewer [20] to evaluate and verify the accuracy of HPV39 E6/E7 reference and mutation sequence generation model. The model was visualized, and the three-dimensional structure of reference and mutation sequence were compared to mark the amino acid mutation site for analyzing whether the gene mutation affected on the three-dimensional structure of the protein or not by PyMOL 2.2.0 [21].

## Results

### The prevalence of HPV39 in Southwest China

**Fig. 2:**
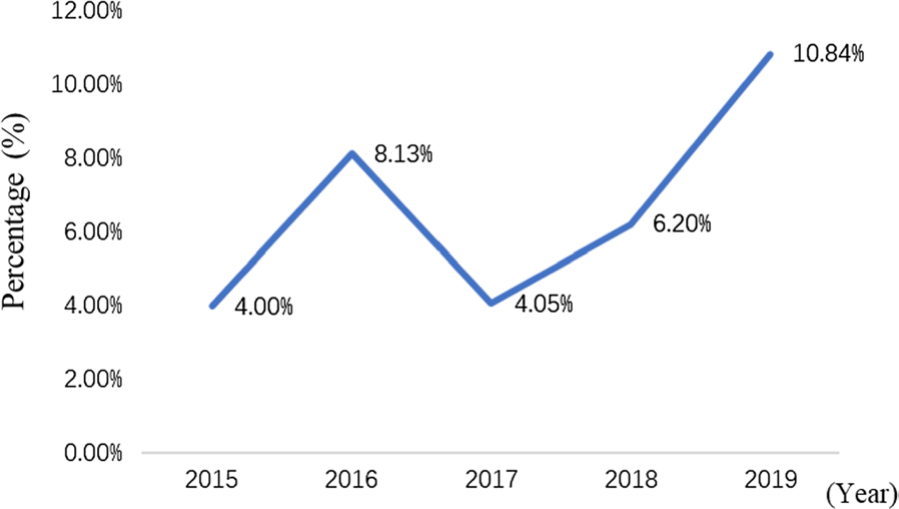
The detection rate of HPV39 in all positive samples during 2015–2019

344 HPV39 cases were detected in all 23,054 samples, including 334 women and 10 men, accounting for 1.49% of the total samples, and 6.02% (344/5718) of HPV positive samples, ranking seventh in HR-HPV detection rate and first in α-7 h-HPV among all positive samples (Fig. 1). The detection rates of HPV39 in all positive samples during recent five years were changed, 4% (39/973) in 2015, 8.13% (67/824) in 2016, 4.05% (71/1752) in 2017, 6.20% (91/1468) in 2018 and 10.84% (76/701) in 2019 (Fig. 2). In 344 HPV39 positive samples, 158 cases (45.93%) were single infection, 186 cases (54.07%) were multiple infection. Among the HPV39 co-infection, mixed infection with HPV6 was the most common (18.49%), followed by HPV16 (12.5%), HPV73 and HPV83 had no mixed infection with HPV39 (Table 4). Age is an important determination of HPV infection risk. Among the HPV39 positive population, 18–28 years old (52.62%) was the highest detection rate age group. The age group of younger than 18 years old was the lowest with 1.16% detection rates (Table 5).

**Table 4:** The detection rates of HPV39 with other HPV types co-infection

Type	Number	Percent (%)	Type	Number	percent (%)
6	71	18.49	33	8	2.08
16	48	12.5	35	8	2.08
52	43	11.2	53	8	2.08
68	35	9.11	31	7	1.82
58	32	8.33	59	7	1.82
51	20	5.21	45	4	1.04
81	20	5.21	43	4	1.04
56	17	4.43	82	2	0.52
42	16	4.17	44	1	0.26
18	13	3.39	73	0	0
66	13	3.39	83	0	0
11	9	2.34

**Table 5:** The HPV detection rates in all age groups among HPV39 positive samples

Age (year)	Number	Percent (%)
< 18	4	1.16
18–28	181	52.62
29–39	100	29.70
40–50	45	13.08
51–61	7	2.03
> 61	7	2.03
Total	344	100

### Sequence analysis of HPV39 *E6/E7/L1* genes

Among the 344 HPV39 positive specimens, 306 (88.95%) *E6-E7* and 298 (86.63%) *L1* sequences were amplified and analyzed successfully. All sequences obtained in this study have been uploaded to the GenBank with accession number as follows: E6-MK340878-MK340882; E7-MK340883-MK340890; L1-MK344658- MK344673.

Compared with the reference sequence M62849, 306 HPV39 *E6-E7* sequences obtained in this study showed that 94.12% (288/306) sequences were variant. 19 single nucleotide mutations (8 in *E6* and 11 in *E7*) were detected, the mutation rate of *E6* was 1.68% (8/477), and *E7* was 3.33% (11/330). Among them, 9 (9/19, 47.37%) were non-synonymous mutations and 10 (10/19, 52.63%) were synonymous mutations. Moreover, the nucleotide mutations of C205T, C211T, A289T, T487C, T558G in *E6* and T633G, G792C, G819A, G851A, A858T, C862A in *E7* have not been reported. In addition, two mutations were observed at 558 sites of *E6,* namely T558C (39pe02, 39pe03) and T558G (39pe12, 39pe13) (Table 2).

Compared with the reference sequence M62849, 298 HPV39 *L1* variant sequence were obtained in 298 HPV39 *L1* sequence (100%, 298/298). 26 single nucleotide mutations were detected, 26.92% (7/26) were non-synonymous mutations and 73.08% (19/26,) were synonymous. In *L1* sequence, C6056T, A6257G, G6306A, T6428C, G6456C, A6479G, T6500C, T6506G, T6569G, C6679G, A6869G, T6710G, A6956G and A6974G have not been reported before. C6903T exists in all obtained sequences, details were shown in Table 3.

### Phylogenetic analysis and selective pressure analysis of HPV39 *E6-E7 *and *L1* sequences

**Fig. 3:**
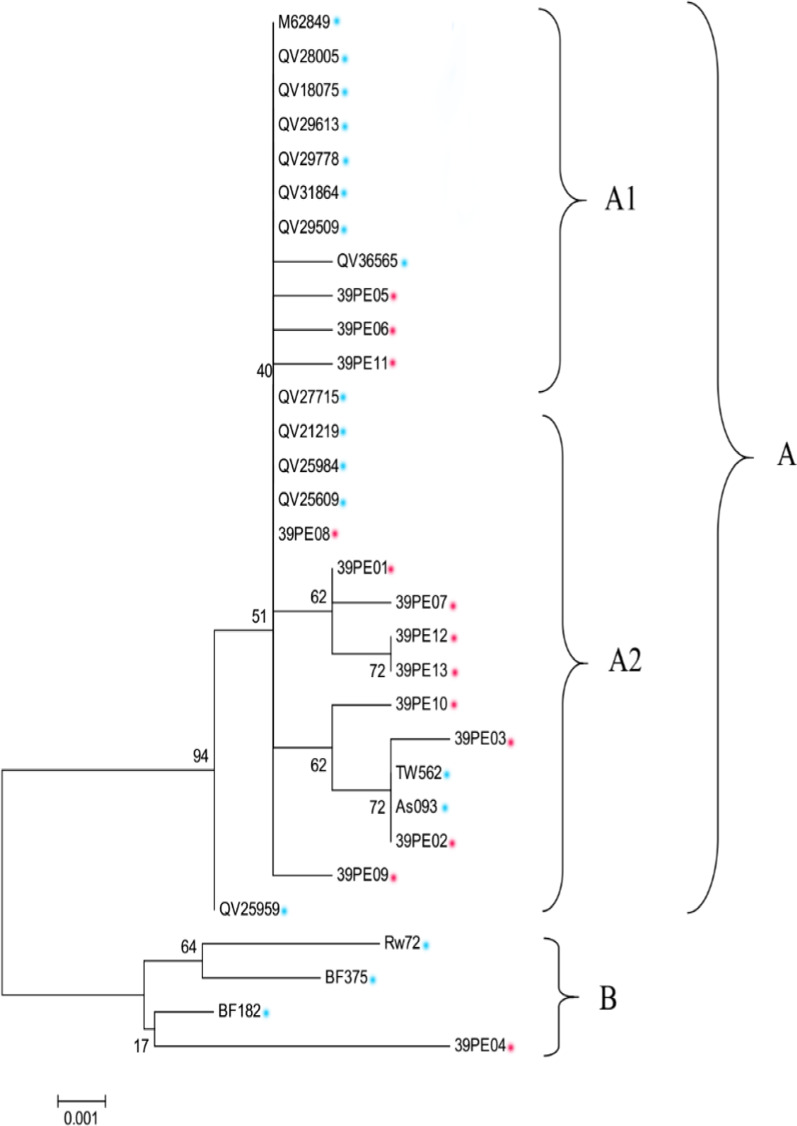
The Maximum-likelihood trees of HPV39 E6-E7

The ML phylogenetic tree of 13 isolates HPV39 *E6-E7* was constructed, which obtained in the study, denoted as 39PE01-39PE13, accompanied by 18 reference sequences. HPV39 variants complete genome sequences were clustered into A (A1 and A2) and B two lineages [20], in our study, only one lineage B variant was detected (Fig. 3). The number of HPV39 isolates sub-lineages A1, A2 and lineage B variants were 58 (28.16%), 141 (68.54%) and 7 (3.39%), respectively. 39PE06 was the most common variant (44.83%, 26/58) in A1 sub-lineage and 39PE02 was the most common variant (17.02%, 24/141) in A2 sub-lineage.

**Fig. 4:**
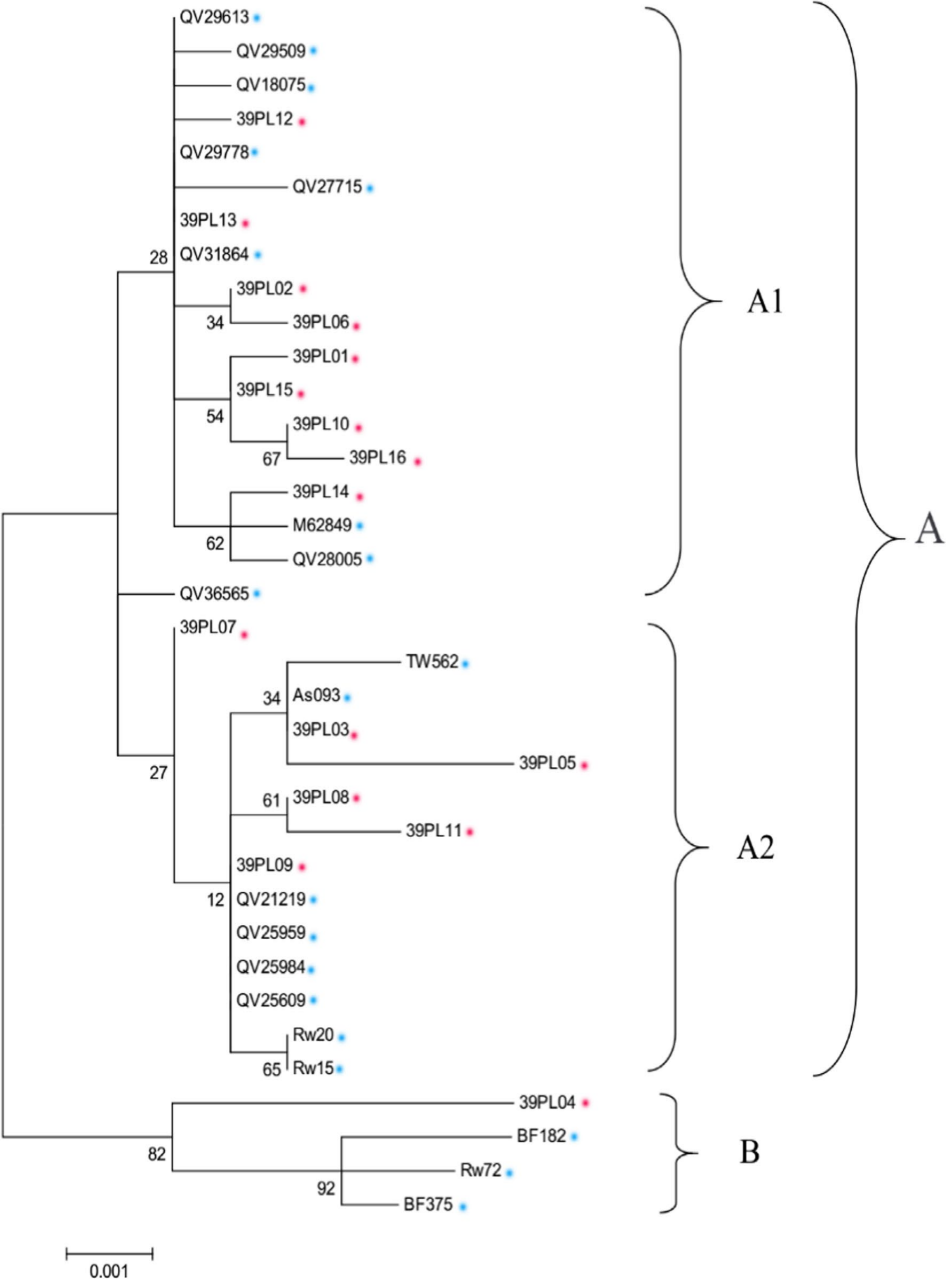
The maximum-likelihood tree of HPV39 L1

The ML phylogenetic tree of 16 isolates HPV39 *L1* was constructed, denoted as 39PL01-39PL16, accompanied by 20 reference sequences (Fig. 4). According to the previously described lineages A (A1 and A2) and B, the ML phylogenetic tree shows the dichotomy of branches [20]. In the A lineage, there were 15 variants, and B lineage was one only. The number of HPV39 *L1* isolates sub-lineages A1, A2 and lineage B variants were 114 (57.58%), 78 (39.39%) and 6 (3.03%), respectively. 39PL02 was the most common variant (21.93%, 25/114) in A1 sub-lineage and 39PL05 was the most common variant (26.92%, 21/78) in A2 sub-lineage.

10 positively selected codon sites and 4 negatively selected codon sites were identified (Tables 6 and 7). Concretely, codon sites 26, 87, 151 in E6 and 75, 180, 222, 272, 284, 346, 356 in L1 are positively selected sites. Codon sites 45, 138, 309, 381 are negative selection sites in the L1 gene.

**Table 6:** HPV39 E6/E7/L1 positive selection site

Positively selected sites (50 significance level)						
Gene	Codon	E[dS]	E[dN]	Normalized E[dN-dS]	Posterior Probability	Bayes Factor
	26	1	10.0336	9.03359	0.929216	419.403
*E6*	87	1	10.0987	9.0987	0.935251	461.476
	151	1	10.7965	9.79648	0.999941	538.503
*E7*	Found no Positively selected sites (50 significance level)					
	75	0.561634	2.3459	1.78427	0.965567	587.849
	180	0.626187	2.25204	1.62585	0.921948	247.618
	222	0.7726	2.24721	1.47461	0.908844	209.008
*L1*	272	0.438175	2.25315	1.81497	0.936722	310.324
	284	0.514167	2.25028	1.73612	0.929747	277.434
	346	0.649058	2.25464	1.60559	0.921272	245.31
	356	0.840996	2.24741	1.40641	0.903727	196.783

**Table 7:** HPV39 E6/E7/L1 negative selection sites

Negatively selected sites (50 significance level)						
Gene	Codon	E[dS]	E[dN]	Normalized E[dN-dS]	Posterior Probability	Bayes Factor
*E6*	Found no negatively selected sites (50 significance level)					
*E7*	Found no negatively selected sites (50 significance level)					
	45	12.7368	0.088334	− 12.6485	0.999999	44,212.9
*L1*	138	12.6934	0.088682	− 12.6048	0.999874	379.199
	309	12.7055	0.088391	− 12.6172	0.999909	526.047
	381	12.7033	0.088331	− 12.615	0.999903	491.748

### The secondary and three-dimensional structure of HPV39 E6/E7 protein

**Fig. 5:**
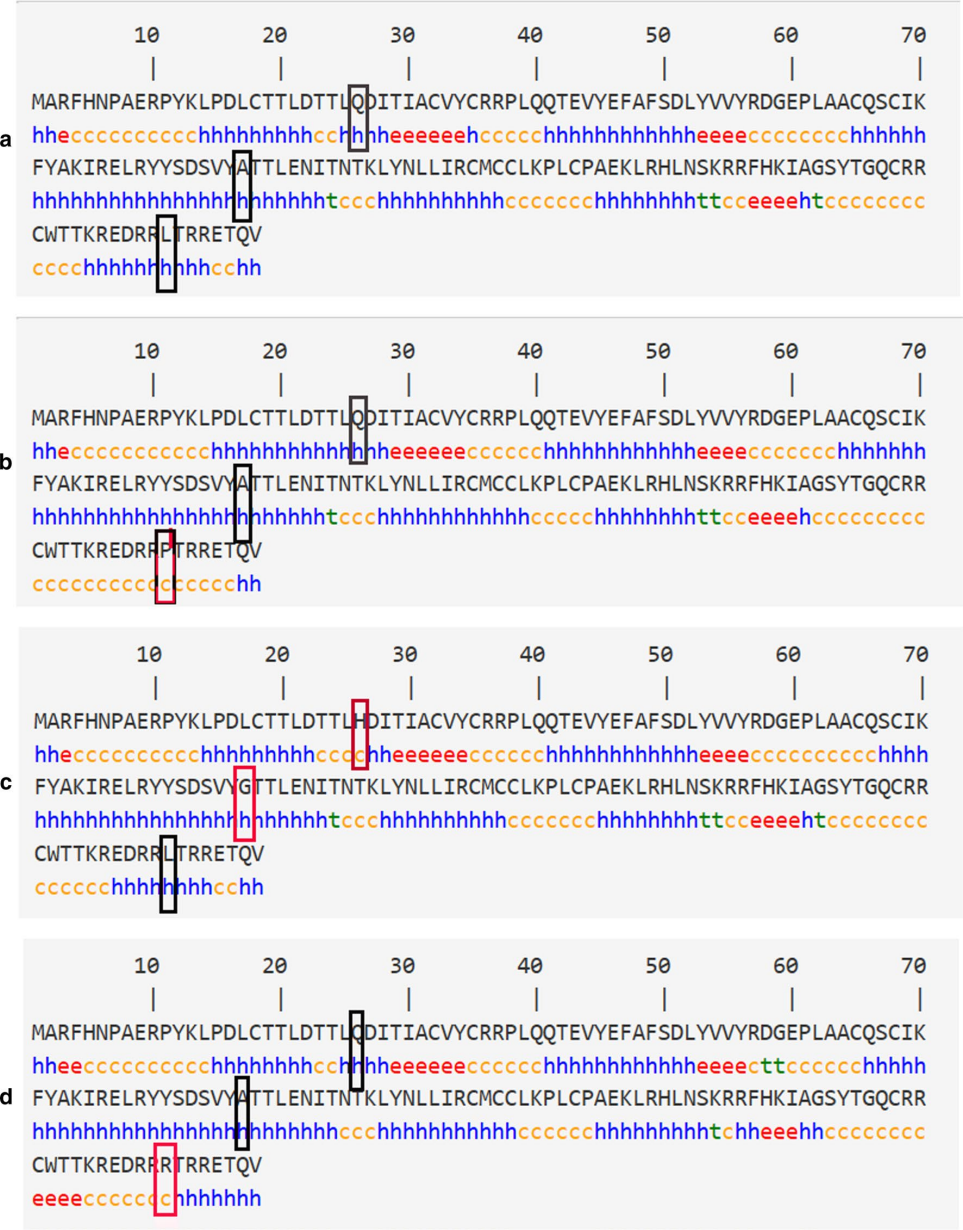
Prediction and comparison results of secondary structure of HPV39 E6

**Fig. 6:**
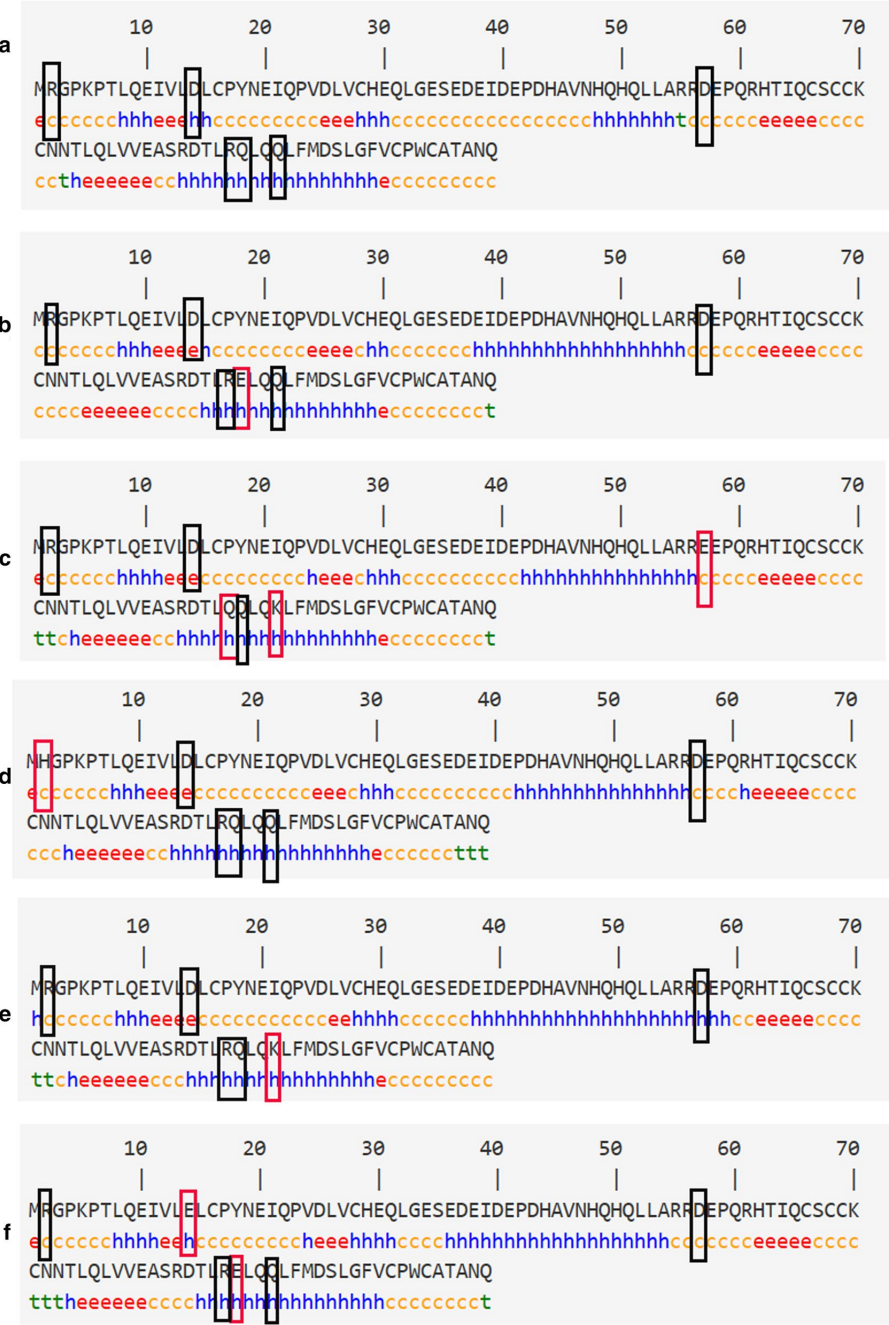
Prediction and comparison results of secondary structure of HPV39 E7

Three E6 variants, 39pe02, 39pe04, 39pe12 and five E7 variants, 39pe02, 39pe04, 39pe05, 39pe09, 39pe10 were detected in our research. The secondary structure of E6 mutation sequence showed that Q26H, L51P and L151R appeared in random crimp (C), and A87G appeared in α-helix (H). Compared with reference sequence M62849, the secondary structure of L151P, L151R and Q26H changed from H to C, and the amino acids substitution also influenced the secondary structure around them (H shortened but C increased), A87G did not change the secondary structure. For HPV39 E7, R2H and D57E appeared in C, D14E, R87Q, Q88E and Q91K appeared in H, and there was no difference with M62849 secondary structure (Figs. 5 and 6).

**Fig. 7:**
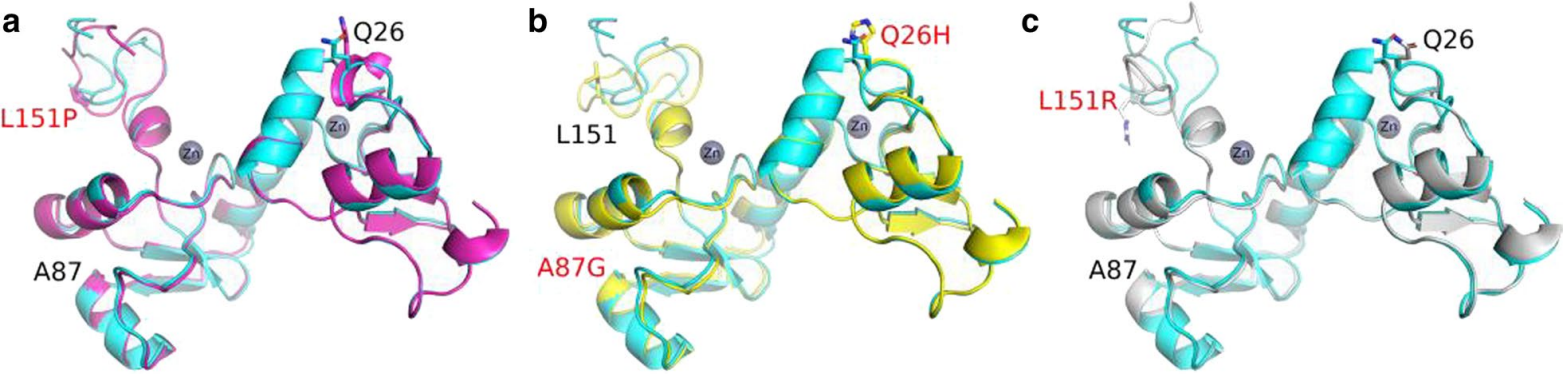
Comparison of HPV39 E6 protein 3D structure prediction models

**Fig. 8:**
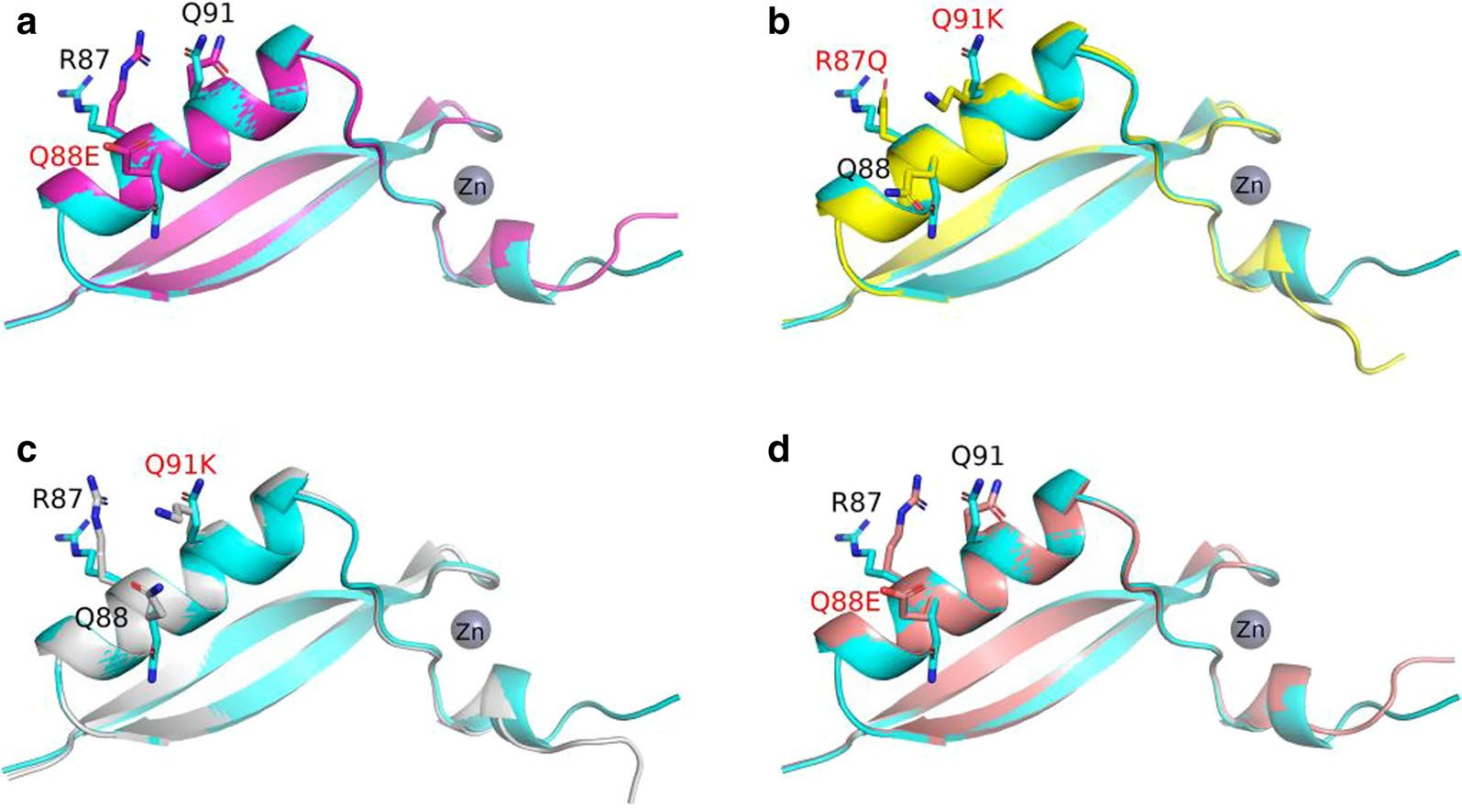
Comparison of HPV39 E7 protein 3D structure prediction models

The three-dimensional structure comparison of E6, E7 reference and mutant protein were shown in Figs. 7 and 8. For E6, the comparison chart of M62849, 39PE02 and 39PE12, L151P/R located in the surface and c-terminal of E6 protein 3D structure, the 151th amino acid substitution may lead the protein more closely to the environment; The comparison chart of M62849 and 39PE04, A87G amino acid substation located in helix, as well as Q26H, they are all exposed to the environment and near to the zinc granule. For E7, the comparison chart of M62849 and HPV39PE02, Q88E located in helix and exposed; The protein structure of M62849 and 39PE04 are difference in 57, 87, 91 position, D57E located in coil and exposed, R87Q and Q91K are both in helix and inside of the protein; The comparison chart of M62849 and HPV39PE10, Q91K is located inside of E7 protein; The comparison between M62849 and 39PE10, D14E located in helix and exposed, while Q88E located in the helix and buried. Non-synonymous mutations somewhat changed the trend of E6/E7 and carboxyl end structural disorder, especially E7.

## Discussion

HR-HPV persistent infection closely related to the occurrence of cervical precancerous lesions and CC [22], its carcinogenicity is mainly realized by E6, E7 oncoproteins encoded by HPV *E6, E7* genes, that lead to genetic instability and cell growth uncontrolled [23, 24]. L1 major capsid proteins can be used to guide the design of prophylactic vaccines and induce the synthesis of high levels neutralizing antibodies major components, virus-like particles (VLP) [25]. HPV prevalence in southwest China during 2014–2019 was studied, demonstrating the significance of HPV39. Considering the relatively high prevalence of HPV39 in southwest China, we selected HPV39 samples to explore the gene polymorphism of *E6, E7* and *L1.*

In this study, 344 HPV39 positive specimens were detected, accounting for 6.01% of the total positive population, higher than that of in Beijing (2.72%), ranking seventh in HR HPV infection rate and first in α-7 HPV; In the past five years, the positive rate of HPV39 was significantly increased, except 2016–2017, all suggested that the prevalence of HPV39 is relatively high in Southwest China and the environment of Southwest China is conducive to the survival of HPV39, so more attention should be paid to the prevention and control of this subtype. The HPV39 multiple infection rate is higher than that of in single infection, may due to the limitation of sample quantity, the different detection categories, and accuracy in different detection methods. Previous studies showed that HPV39 and HPV68 had the most mixed infection [26], but in our study, was HPV39 and HPV6 (18.49%), the probability of mixed infection with HPV68 was 9.11%, ranking fourth, above may due to HPV mixed infection prevalence in different regions was slightly different. Age is an important HPV infection affect factor, previous study showed that 20–24 and 50–54 years old are the two HPV detection peaks age group. The first peak may due to the relatively active sexual lives of women in this age group. The change of female hormone secretion may result in the second positive peak, with the physiological and immune dysfunction, that leads to the resurrection of latent HPV infection and reduced ability to self-return after HPV infection [3]. In our study, HPV39 infection rate was the highest in 18–28 years old, consistent with the previous study results, indicating that age is an important HPV infection rate affecting factor. However, the second peak did not appear in Southwest China, that possibly because the above two infection peaks were the statistical analysis results of all HPV-positive patients, while this study only focuses on the statistical analysis of HPV39 positive patients, fewer age 50–54 participants participate in this study and the high age group is not the HPV39 susceptible population, resulting in the peak age of HPV39 positive patients aged 50–54 years old lacking.

HPV *E6, E7* and *L1* gene of 344 genital secretion samples were sequenced to analyze the gene variation, the distribution of HPV39 mutant and the effect of non-synonymous mutation on protein structure and function in this area. Our study showed that *E7* (3.33%) had the highest mutation rate, followed by *L1* (1.71%) and *E6* (1.68%), indicating that *E6* may be a more suitable target for HPV therapeutic vaccine than *E7* and *L1;* Inconsistent with previous research results that *E7* is a more suitable target for HPV therapeutic vaccines [13, 27], that may due to the target of HPV therapeutic vaccine is different in different HPV types and the limitation of sample size. In the HPV39 *E6, E7* and *L1* sequence, 29 (5 in *E6*, 8 in *E7* and 16 in *L1*) mutation sequences were detected, comparing with reference sequence of *E6* (mk340878-mk340882), *E7* (mk340883-mk340890) and *L1*(mk344658-mk344673). The maximum variation of *E6, E7* and *L1* gene in the positive samples was 1.05% (5/477), 1.52% (5/330) and 0.92% (14/1518) respectively, inconsistent with previously reported in other regions (0.63%, 0.91%, 1.12%), suggesting that the variation in HPV39 *E6, E7* and *L1* might be related to the distribution of regions and populations with regional characteristics.

HPV39 *E6, E7* and *L1* gene belongs to A1, A2 and B lineages, and A pedigree outstand (94.45% for *E6-E7*; 94.63% for *L1*) suggesting A pedigree should be emphasized in the development of HPV39 vaccine, HPV39 B-pedigree is rare, and no B-pedigree mutant were reported previously in China. In this study, 39pe04 and 39pl04 two B-pedigree mutation sequences were isolated and pathogenicity of B-pedigree was stronger than that of A-pedigree, suggesting that more attention should be paid to the prevalence and prevention of B-pedigree HPV39 in the future.

Positive site selection may affect the virus infection efficiency and immunogenicity. The positive selection sites in E6 are 26, 87, 151, and in L1 are 75, 180, 222, 272, 284, 346, 356, no positive selection sites in E7 were detected, the positive selection sites may play an important role in improving the survival ability of HPV39 (e.g., resistance to immune response) and adapting to evolution. The codons 45, 138, 309 and 381 in L1 are negative selection sites, no mutation site was detected in this study, suggesting that mutation at these sites may harmful to the virus survival and evolution, so more attention should be pay to the mutation effect of these sites in the future research.

E6 and E7 are main carcinogenic protein and plays an important role in cervical lesions. Amino acid replacement may affect the carcinogenic potential of E6 and E7 protein and the biological functions of protein were mainly determined by its secondary and three-dimensional structure. The secondary structures of L151P (T558C), L151R (T558G) and Q26H (G596A) in E6 were changed from H to C by comparing with the reference sequence. These amino acid substitutions also have some effect on the secondary structures around them (H shortened and C increased). 145–149 were PDZ domain-containing combined regional that was the target of E6 proteins for cellular transformation, crystal structures reveal that 152R-R-R-E-T-Q-V158 of E6 peptide are involved in PDZ recognition [28, 29]. The regions in the half of carboxy terminal is mainly involved in the p53 binding [30], the L151P, L151R of HPV39 located in the surfaces of HPV E6 and close to the PDZ binding, recognition region that probability influence the function of P53 binding and degradation. E6 has four Cys-X-X-Cys repetitions, respectively located amino acid residues 32–35, 65–68, 105–108 and 138–141, Cys-X-X-Cys motifs have been suggested to coordinate metal binding and are frequently found in nucleic acid binding proteins [31]. A87G and Q26H close to the Zinc, those mutation may influence the protein binding and degradation. Meanwhile 26, 87, 151 are the positive selection sites in E6 and play an important role in improving the survival ability of HPV39, the positive selection sites screening results also verified the effects of the above mutations on protein function to some extent. For HPV39 E7, R2H and D57E appeared in C, D14E, R87Q, Q88E and Q91K appeared in H, and there was no difference with M62849 secondary structure. HPV E7 protein was divided into three main domains CR (conserved region)1, aa 1–15; CR2, aa 16–37; and CR3, aa 38–98, CR1 and CR2 mediate binding of viral oncoproteins to overlapping cellular proteins, including the product of retinoblastoma gene (pRB), cyclin A and cyclin E [32, 33]. 21–29 associated with the binding of retinoblastoma (Rb) tumor suppressor, since E7 acts as the major immortalizing protein through Rb/E2F pathway, amino acid alternations in Rb binding domain may lead to the change of E7′s ability to immortalize cells among variants [34]. The CR3 domain contains two “Cys-X-X-Cys” sequences located at amino acid residues 58–61 and 91–94 participating in Zn binding, are the most important regions associated with the transforming ability of E7 protein [32, 35]. The zinc finger structure at the C-terminal was reported essential for HPV16 E7 to stabilize its structure and biological function, the disruption of the 91Cys-X-X-Cys94 motif that lies towards the carboxyl terminus of E7 protein appeared to result in a greatly impaired transforming ability and reduced transactivation [32, 35]. The mutations in 91th amino-acid caused the loss of the zinc-binding CR3 domain of E7, resulting in a highly unstable protein that can be quickly degraded, and generating more epitopes to be presented in the MHCI pathway, so does the Q91H [36]. R2H located in metal binding motifs, the transformation of E7 has been demonstrated to be eliminated by the mutation in 2th amino acid [35]. D14E located in CR1, mediate protein binding; D57E, R87Q, Q88E close to 58Cys-X-X-Cys61 and 91Cys-X-X-Cys94, that participating in Zn binding, the amnio acid substitution may influence the transforming ability of the E7 protein. These non-synonymous mutations slightly change the E6/E7 amino terminal and the trend of the carboxyl end structure disorder, A certain degree of secondary and three-dimensional structure dislocation were existed between the reference and mutant sequence. Structure changes may lead to the different binding ability to the host p53, RB protein and other potential proteins, that may affect the pathogenicity of HPV39. Therefore, it is necessary to study the function of these non-synonymous mutations furtherly.

This study enrichment the data of HPV39 gene polymorphism and evolution in Chinese population, as well as provide a molecular biological basis for the future research analysis of HPV39 E6/E7/L1 and the design of therapeutic vaccine. However, due to the sample size limitation, the result of this study still has some shortcoming. Therefore, it is necessary to carry out multi-region and large sample population research, conduct a series of cell experiments to verify the above mutation sites function, explore the relationship between HPV39 gene polymorphism and genital lesions as well as its action mechanism.

## Conclusion

HPV39 is a high prevalence HPV type in Southwest China, which is prone to mixed infection with other HPV types, especially with HPV6. 18–28 years old is the highest HPV infection rate age group. A pedigree HPV39 *E6, E7* and *L1* gene is dominant in southwest china, two B-pedigree mutant sequences were isolated and its pathogenicity was stronger than A-pedigree. Positive selection sites may affect the virus infection efficiency, immunogenicity and improve the survival ability of HPV39 as well as adaptation to evolution. Negative selection sites suggesting that mutation at these sites may harmful to the virus survival and evolution. A certain degree of dislocation was existed between the protein secondary and three-dimensional structure of the reference and mutant sequence. The changes of structure may lead to the different binding ability to the host p53 protein and other potential proteins, thus affect the pathogenicity of HPV39.

## Data Availability

All data generated or analyzed during this study are included in this article and GeneBank.

## Abbreviations

CC: Cervical cancer
CR: Conserved region
VLP: Virus-like particles
pRB: Retinoblastoma gene
HPV: Human papillomavirus
dsDNA: Double stranded DNA
MHC: Major histocompatibility complex
HR-HPV: High-risk human papilloma virus
MEGA 6: Molecular Evolutionary Genetics Analysis version 6.0
